# Development of a SARS‐CoV‐2 neutralization assay based on a pseudotyped virus using a HIV system

**DOI:** 10.1002/mco2.517

**Published:** 2024-03-22

**Authors:** Ziteng Liang, Jincheng Tong, Xi Wu, Shuo Liu, Jiajing Wu, Yuanling Yu, Li Zhang, Chenyan Zhao, Qiong Lu, Jianhui Nie, Weijin Huang, Youchun Wang

**Affiliations:** ^1^ Chinese Academy of Medical Sciences & Peking Union Medical College Dongcheng District, Beijing China; ^2^ Division of HIV/AIDS and Sex‐transmitted Virus Vaccines, Institute for Biological Product Control, National Institutes for Food and Drug Control (NIFDC), WHO Collaborating Center for Standardization and Evaluation of Biologicals NHC Key Laboratory of Research on Quality and Standardization of Biotech Products and NMPA Key Laboratory for Quality Research and Evaluation of Biological Products Beijing China; ^3^ Changping Laboratory Changping District, Beijing China; ^4^ Beijing Yunling Biotechnology Co., Ltd. Beijing China

**Keywords:** fluorescent pseudovirus, HIV system, neutralization assay, SARS‐CoV‐2

## Abstract

Regarding the extensive global attention to severe acute respiratory syndrome coronavirus 2 (SARS‐CoV‐2) that constitutes an international public health emergency, pseudovirus neutralization assays have been widely applied due to their advantages of being able to be conducted in biosafety level 2 laboratories and having a high safety factor. In this study, by adding a blue fluorescent protein (AmCyan) gene to the HIV system pSG3‐△env backbone plasmid *Hpa*I and truncating the C‐terminal 21 amino acids of the SARS‐CoV‐2 spike protein (S), high‐titer SARS‐CoV‐2‐Sdel21‐AmCyan fluorescent pseudovirus was successfully packaged. The fluorescent pseudovirus was used to establish a neutralization assay in a 96‐well plate using 293T cells stably transfected with the AF cells. Then, parameters such as the ratio of backbone and membrane plasmid, sensitive cells, inoculation of cells and virus, as well as incubation and detection time were optimized. The pseudovirus neutralization assay demonstrated high accuracy, sensitivity, repeatability, and a strong correlation with the luminescent pseudovirus neutralization assay. Additionally, we scaled up the neutralizing antibody determination method by increasing the plate size from 96 wells to 384 wells. We have established a robust fluorescent pseudotyped virus neutralization assay for SARS‐CoV‐2 using the HIV system, providing a foundation for serum neutralization antibody detection, monoclonal antibody screening, and vaccine development.

## Graphical abstract

Regarding the extensive global attention to SARS‐CoV‐2 that constitutes an international public health emergency, pseudovirus neutralization assays have been widely applied due to their advantages of being able to be conducted in biosafety level 2 laboratories and having a high safety factor. In this study, by adding a blue fluorescent protein (AmCyan) gene to the HIV system pSG3‐△env backbone plasmid *Hpa*I and truncating the C‐terminal 21 amino acids of the SARS‐CoV‐2 spike protein (S), high‐titer SARS‐CoV‐2‐Sdel21‐AmCyan fluorescent pseudovirus was successfully packaged. The fluorescent pseudovirus was used to establish a neutralization assay in a 96‐well plate using 293T cells stably transfected with the ACE2 receptor and Furin protease (AF cells). Then, parameters such as the ratio of backbone and membrane plasmid, sensitive cells, cell addition, virus inoculation, incubation time, and detection time were optimized. The pseudovirus neutralization assay showed good accuracy, sensitivity, repeatability, and a good correlation with the luminescent pseudovirus neutralization assay. Additionally, we further developed a high‐throughput and automated 384‐well plate neutralization assay based on the 96‐well plate. In conclusion, we have established a robust fluorescent pseudovirus neutralization assay for SARS‐CoV‐2 using the HIV system, providing a foundation for serum neutralization antibody detection, monoclonal antibody screening, and vaccine development.

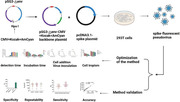

## 1 INTRODUCTION

The severe acute respiratory syndrome coronavirus 2 (SARS‐CoV‐2) appeared at the end of 2019 and caused a large‐scale outbreak of an acute respiratory disease called Corona Virus Disease 19 (COVID‐19).^1^, ^2^ This new human coronavirus, known as SARS‐CoV‐2, has swept across the globe and has been declared an international public health emergency by the World Health Organization.^3^ As of June 2023, there have been a staggering 768 million confirmed cases of COVID‐19 worldwide, emphasizing the critical necessity of creating a vaccine or medication to combat SARS‐CoV‐2. However, the speed of research is greatly limited by the requirement of a biosafety level‐3 laboratory (BSL‐3) for cultivating authentic viruses. In response to this issue, neutralization assays using pseudoviruses can be conducted in biosafety level‐2 laboratories (BSL‐2). Pseudoviruses only can infect cells once and have the advantages of being safe and efficient.^4^, ^5^

SARS‐CoV‐2 belongs to the family Coronaviridae, the subfamily Coronavirinae, and the genus Coronavirus. It is a single‐stranded positive‐sense RNA virus that has four structural proteins: S, M, E, and N, respectively, the spike protein, membrane protein, envelope protein, and nucleocapsid protein. Among them, the spike protein S is the most extensively studied^6^, ^7^ and the most important structural protein.^8^ It binds with angiotensin‐converting enzyme 2 (ACE2) on the cell surface, facilitating viral membrane fusion and entry into the cell.^9^, ^10^, ^11^, ^12^, ^13^ Studies have indicated that the role of furin protease in the cleavage of the spike protein is crucial..^14^, ^15^, ^16^ After mutation of the furin cleavage site at position 814, the fusion ability of the wild‐type S protein is weakened.^17^ Therefore, furin plays a crucial role in SARS‐CoV‐2 infection. Based on the above research, we stably transfected the ACE2 receptor and furin protease on 293T cells and established a stably transfected cell line named AF. Some research has shown that truncation modification of the spike protein can significantly improve the packaging titer of pseudovirus.^3^, ^18^, ^19^, ^20^ Combined with our laboratory's findings on the truncation modification of the S protein, truncating 21 amino acids from the C‐terminus of the S protein can effectively enhance the packaging efficiency of pseudovirus.

When exogenous pathogens invade the body, it stimulates the production of protective substances,^21^ which can prevent the entry and infection of viruses into cells by reducing the binding of viruses to cell surface epitopes. These substances are defined as neutralizing antibodies,^22^ and the level of neutralizing antibodies is an essential indicator of the protective effect of vaccine immunity and an important basis for vaccine evaluation and quality control.^23^ Currently, established SARS‐CoV‐2 neutralizing antibody test based on 96‐well plates include plaque reduction neutralization tests (PRNT),^24^ chemiluminescence Fluc pseudovirus neutralization assays, and green fluorescent protein (GFP) pseudovirus neutralization assays based on the VSV system.^4^, ^5^ PRNT is based on the authentic virus in a BSL‐3 laboratory and is limited by a long detection period.^4^ It is complex to detect the Fluc pseudovirus neutralization assay using the VSV system, as it requires external substrates and is expensive. Pseudovirus using the VSV system GFP fluorescence, although the external substrate is avoided, the problem of residual VSV needs to be considered. Therefore, developing an HIV system of fluorescence pseudotyped virus neutralization assay is essential. This method combines the advantages of rapid and simple fluorescence pseudovirus neutralization assay while avoiding the hazards of authentic virus operation in PRNT. It provides technical assistance for neutralizing antibodies in serum and screening antiviral drugs. For the screening of large samples, the fluorescence pseudovirus neutralization assay based on the 384‐well plate has been widely studied due to its small sample volume, automation, and high throughput.

In this study, based on the HIV system, the pSG3‐△env backbone plasmid with AmCyan gene was added, and the expression of the fluorescent protein was enhanced by adding the CMV promoter and Kozak sequence before the fluorescent reporter gene. After truncating 21 amino acids in the tail of the spike, we obtained higher titers of the fluorescent pseudotyped virus, and stable HIV system 96‐well plate and 384‐well plate neutralizing antibody assays were established.

## 2 RESULT

### 2.1 Optimization of the method

#### 2.1.1 The ratio of backbone plasmid and membrane plasmid

**FIGURE 1:**
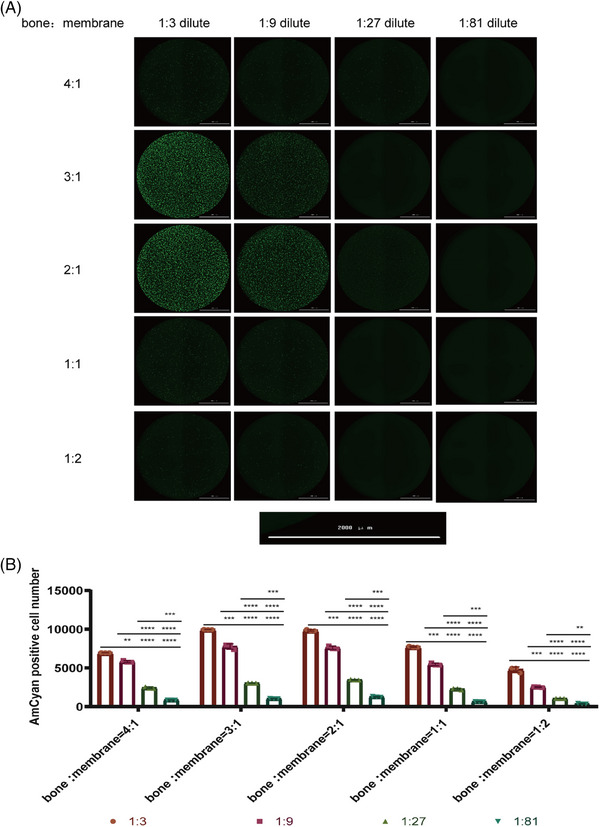
Optimization of the ratio of backbone plasmid and membrane plasmid. (A) After threefold gradient dilution, titration of SARS‐CoV‐2‐D614Gdel21‐AmCyan fluorescent pseudovirus. The scale bar is 2000 µm. (B) The AmCyan‐positive cell number was detected at different ratio of backbone plasmid and membrane plasmid.

To package out high titers of fluorescent pseudoviruses, we explored the ratio of backbone plasmids and membrane plasmids in six‐well plates (Table 1 and Figure 1A) and the total amount of plasmids per well was 6 µg. The fluorescent pseudovirus was diluted by a threefold gradient, and the number of AmCyan‐positive fluorescent cells was detected after 36 h. The counts of AmCyan‐positive points are higher when backbone plasmid and membrane plasmid = 2:1, so the ratio of backbone plasmid and membrane plasmid is 2:1 (Figure 1B).

**TABLE 1:** The ratio of the addition amount of backbone plasmid to membrane plasmid.

Group	Bone:Membrane	Bone (µg)	Membrane (µg)
1	Bone:Membrane = 4:1	4.8	1.2
2	Bone:Membrane = 3:1	4.5	1.5
3	Bone:Membrane = 2:1	4	2
4	Bone:Membrane = 1:1	3	3
5	Bone:Membrane = 1:2	2	4

#### 2.1.2 Cell tropism, detection time, cell addition, virus inoculation, and incubation time

**FIGURE 2:**
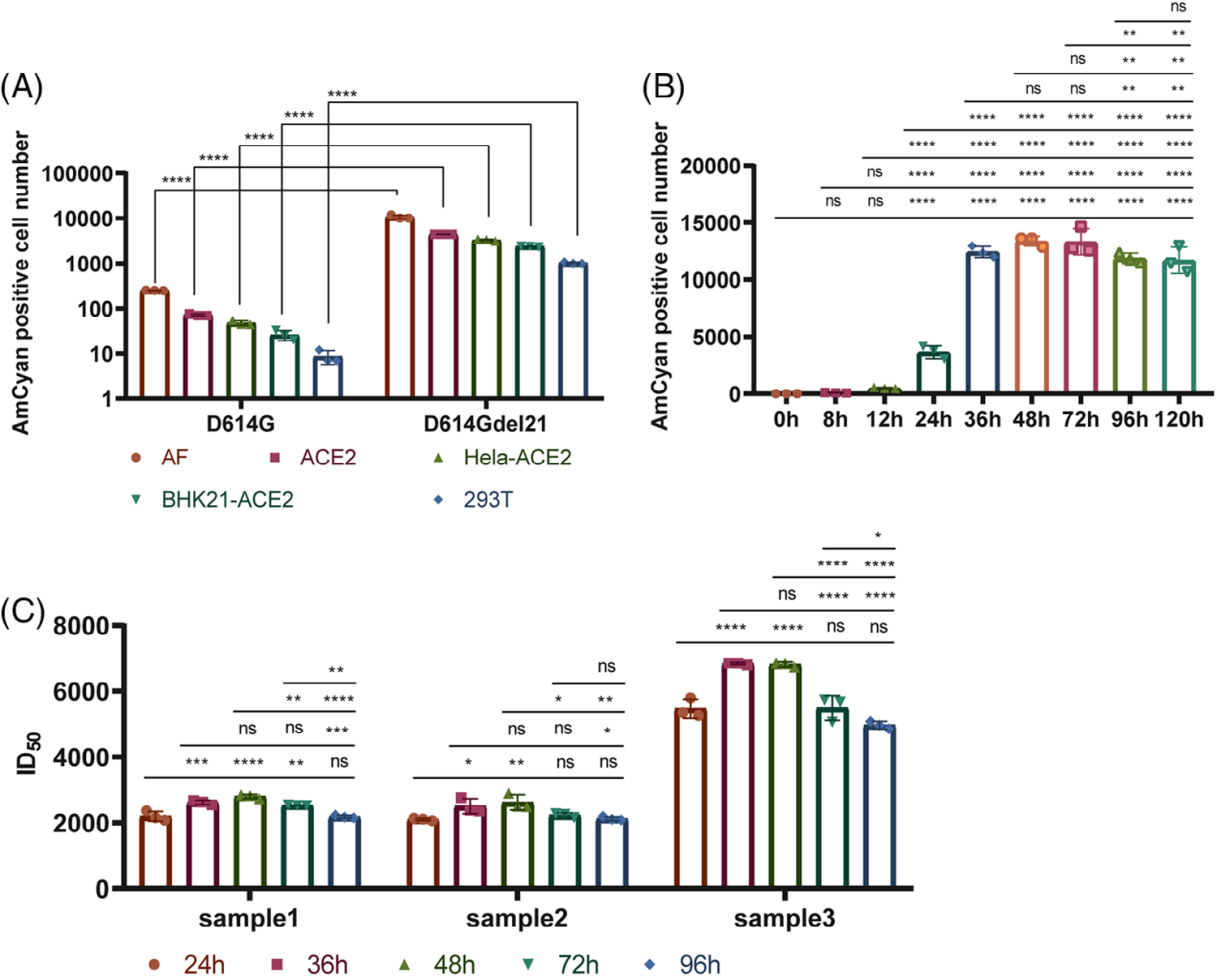
Optimization of the selection of sensitive cells and detection time. (A) A comparison between the infection efficiency of SARS‐CoV‐2‐D614Gdel21‐AmCyan and SARS‐CoV‐2‐D614G‐AmCyan is conducted on AF, ACE2, Hela‐ACE2, BHK21‐ACE2, and 293T cells. (B) The test on pseudovirus titration compares the number of AmCyan‐positive cells detected at 0, 8, 12, 24, 36, 48, 72, 96, and 120 h. (C) In the pseudovirus neutralization test, the ID50 of sample 1−3 is compared at 24, 36, 48, 72, and 96 h.

In order to ascertain the cells that are sensitive, we conducted additional research on the infectivity of D614G and D614Gdel21 fluorescence pseudoviruses in different cell lines (293T cells, AF cells, ACE2 cells, Hela‐ACE2 cells, and BHK21‐ACE2). Our findings revealed that AF cells exhibited the maximal number of AmCyan‐positive cells, followed by ACE2 (Figure 2A). Then, we discovered the ACE2 protein was consistent in the five cells (Figure S1A) through western blotting assay. Based on this, AF cells are the most sensitive cells due to the additional furin protease. After truncation, the titers of the SARS‐CoV‐2 pseudotyped virus were 65‐fold than before. By measuring the expression of D614G and D614Gdel21 spike proteins in 293T cells (Figure S1B), we found that truncating 21 amino acids could enhance the expression of the spike protein, which was previously found in the 18 amino acid truncation.^25^ The observed high infectivity might be attributed to the enhanced spike expression.

In order to determine the most suitable time for detection, we observed the quantity of AmCyan‐positive cells at 0, 8, and every 12 h after the incubation period in a titration experiment (Figure 2B). In the neutralization assay, we monitored three representative samples: sample 1 is a monoclonal antibody specifically targeting SARS‐CoV‐2; sample 2 is a serum from COVID‐19 convalescent; sample 3 is a SARS‐CoV‐2‐positive serum from a guinea pig, and measured the ID50 values at different times (Figure 2C). The titration assay results showed no fluorescence before 12 h, with a rapid increase in positive cells between 24 and 48 h after infection, reaching a plateau within 48 h and remaining stable between 36 and 60 h. The ID50 values of samples remained relatively stable at 36−48 h, as indicated by the neutralization assay results at different time points. Thus, quantifying the infective performance and neutralization effect of pseudotyped viruses can be achieved by counting the AmCyan‐positive cells at 36 h.

**FIGURE 3:**
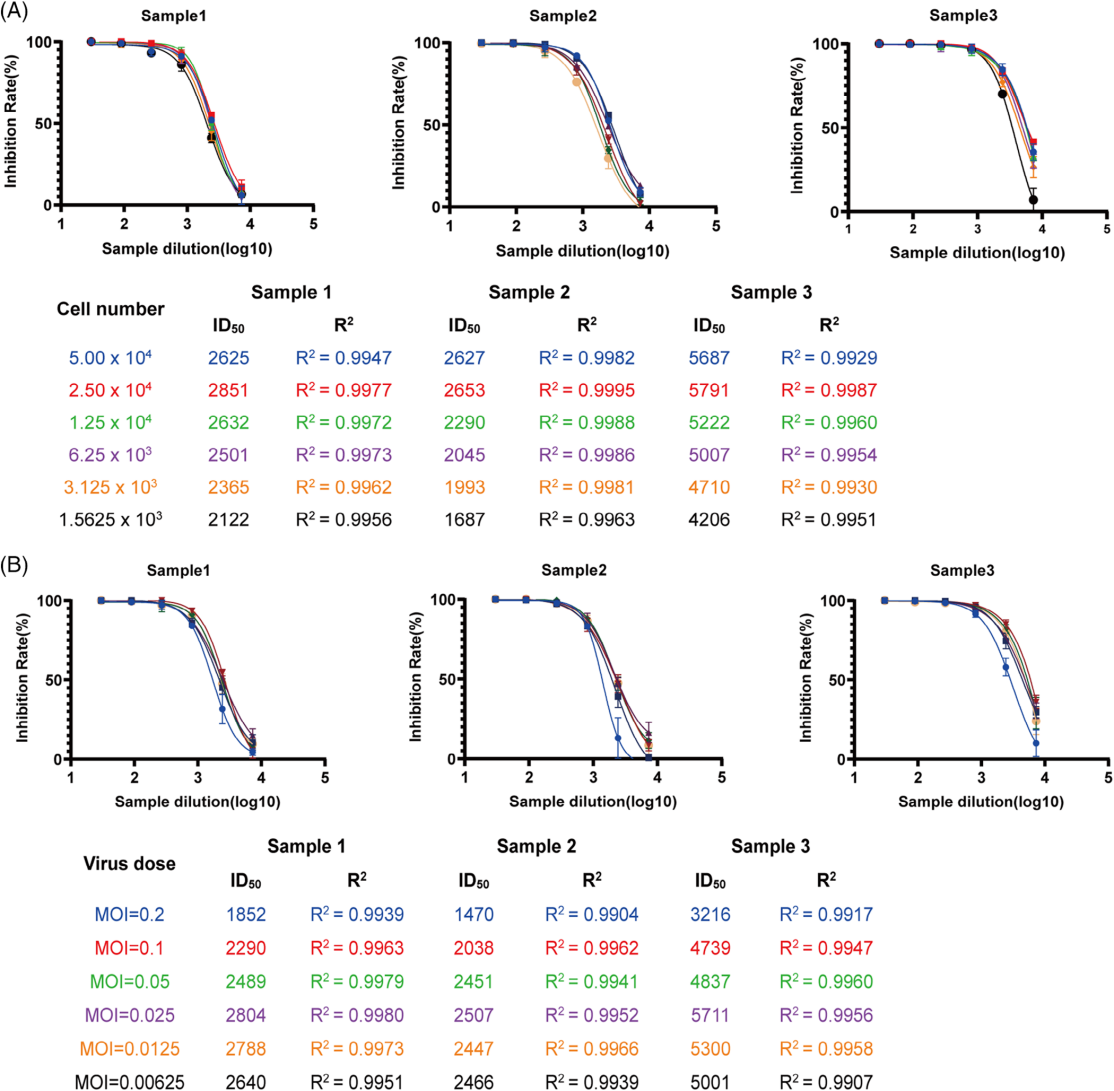
The optimization process for cell addition and virus inoculation. (A) In order to optimize the seeding cell number, a range of 1.5625 × 103 to 5 × 104 cells/well were added, and the cells were then infected with an inoculant dose of 0.025 MOI. The ID50 values of sample 1−3 were determined using nonlinear regression, specifically the inhibitor vs response method. (B) The optimization process for inoculant dose of the pseudovirus was investigated using doses ranging from 0.00625 to 0.2 MOI. The amount of cells added was 2.5 × 104 cells/well, and the ID50 values were calculated using nonlinear regression, again utilizing the inhibitor versus response method. The experimental data obtained from three repeated trials.

In order to determine the suitable cell addition, neutralization experiments were conducted by introducing a range of cells/well from 1.5625 × 103 to 5 × 10^4^. An MOI of 0.0125 was used for adding the virus. The findings illustrated that the maximum count of AmCyan‐positive cells occurred at a concentration of 2.5 × 104 cells/well (Figure 3A). The inhibition curve for the three samples was calculated at various dilutions (log_10_), and the *R* ^2^ values were greater than 0.9, indicating a good fit of the curve combination. Based on these findings, 2.5 × 104 cells/well were selected as the best cell addition.

In order to identify the suitable virus addition, we tested different virus concentrations, with a minimum MOI of 0.00625 and a maximum MOI of 0.2. We maintained the number of cells added at 2.5 × 104 cells/well for the pseudotyped virus neutralization test. We plot the sample inhibition at different dilutions (Figure 3B). The *R* ^2^ values for all the curves were more significant than 0.9, indicating a good fit. The ID50 values of the sample showed a stable tendency when the virus MOI was set at 0.025. As a result, we determined that the suitable amount of virus to be added is MOI = 0.025, which was approximately 800 cells positive for AmCyan.

**FIGURE 4:**
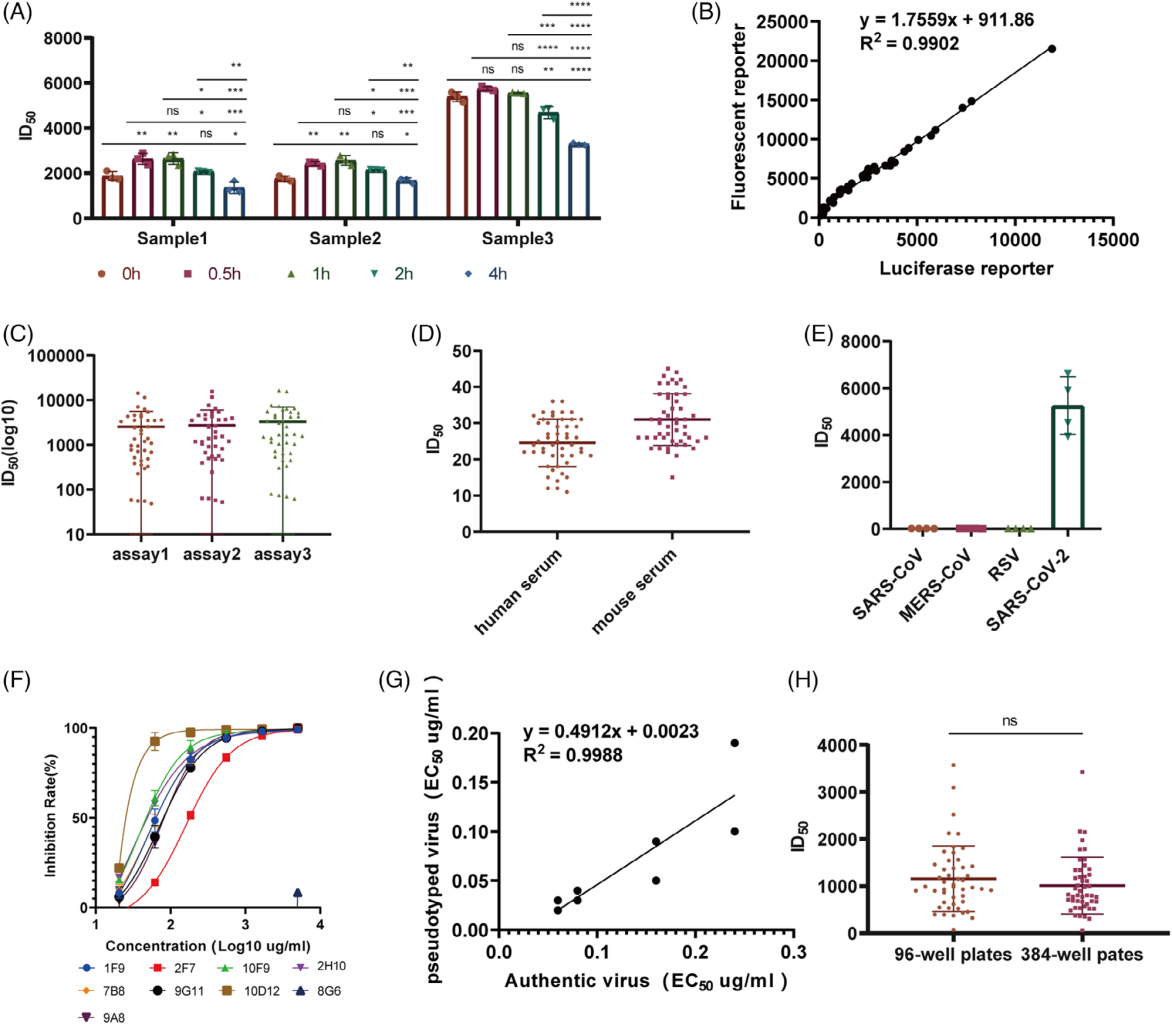
Methodological validation and applications. (A) The neutralization test results of fluorescent pseudovirus were assessed under the condition that the number of cells added was 2.5 × 104 cells/well and the virus inoculation amount was MOI = 0.0125. (B) Correlation of the 96‐well plate fluorescent pseudovirus neutralization test and the 96‐well plate Fluc pseudovirus neutralization test. (C) Sensitivity of the pseudovirus neutralization assay. (D) Repeatability of the pseudovirus neutralization assay. (E) Specificity of the pseudovirus neutralization assay. (F) Using the established fluorescent pseudovirus neutralization assay to screen nine of monoclonal antibodies. (G) The correlation between EC50 values from the 96‐well pseudovirus neutralization assays and the authentic neutralization assay was analyzed. The fitted regression line equation was *y* = 0.4912*x* + 0.0023, with an *R* ^2^ value of 0.99. (H) The ID50 values of 42 human serum samples were obtained to analyze the consistency of both the 384‐well and 96‐well plate fluorescent pseudovirus neutralization assays.

In order to identify the suitable incubation time, we tested the amount of AmCyan‐positive points within the range of 0–4 h in the neutralization assay with the fluorescent pseudovirus samples at 37°C (Figure 4A). The results indicated no noticeable difference in ID50 values when the incubation time ranged from 0.5 to 1 h. However, when the incubation time exceeded 2 h, the ID50 values decreased. Therefore, we concluded that the suitable incubation time was between 0.5 and 2 h, and for subsequent experiments, we chose the incubation time of 1 h.

### 2.2 Method validation

#### 2.2.1 Linear regression analysis

Comparing the HIV system fluorescent pseudovirus neutralization test established in this study with the existing VSV system SARS‐CoV‐2‐Spike‐Fluc pseudovirus neutralization test. We selected 42 guinea pigs positive sera as test samples and used the ID_50_ values obtained by the two neutralization test methods to perform linear regression analysis (Figure 4B). The linear equation is *y* = 1.7559*x* + 911.86 *R* ^2^ = 0.9902 (X: VSV chemiluminescence system, Y: HIV fluorescence system), thus indicating a reasonable correlation between the two methods.

#### 2.2.2 Accuracy and specificity

We validated the accuracy by spiked recovery assays containing a mAb and a human‐negative serum. The spiked recovery was calculated by mixing onefold, fivefold, and 25‐fold in negative serum for the ID50 value. The recovery rates of 110.5, 99.85, and 100.7% for the three respective additions, thus suggesting a reasonable accuracy.

To prove the specificity of this method, we selected four SARS‐CoV positive sera, four MERS‐CoV positive sera, four RSV positive sera, and four SARS‐CoV‐2‐positive sera for neutralization test, and their ID_50_ value was calculated (Figure 4E). Only SARS‐CoV‐2‐positive serum can specifically neutralize the pseudotyped virus. Thus, this method has reasonable specificity.

#### 2.2.3 Sensitivity and repeatability

In order to obtain the cutoff values for the neutralization method, a total of 100 negative sera, including 50 human serum samples and 50 guinea‐pig serum samples, were used. During the testing, the serum underwent a twofold initial dilution, followed by a serial twofold gradient dilution to determine its ID50 value (Figure 4C). The results showed that the mean of human negative serum was 24.59, the SD was 6.50, the mean of guinea pig negative serum was 30.98, and the SD was 7.11. The cutoff values were determined as the mean plus 1.96 SD, resulting in a detection limit of human serum of 37.33 and 44.92 for guinea pig serum. Therefore, the cut‐off values were ultimately established as 40 and 50, respectively. Following calibration against the NIBSC code: 20/136, 1000 IU/mL, the corrected results are 20.5 and 25.6 IU, respectively. In addition, the specificity is 100%.

To prove the repeatability of this method, we selected 20 guinea pig sera immunized with three injections of spike protein, detected the ID_50_ of neutralizing antibody titers with D614G pseudovirus, and repeated the detection three times at different times. The detection method showed good repeatability as seen in repeated detection (Figure 4D), with intraassay CV values ranging from 0.04 to 14.2% and interassay variability CV values ranging from 4.20 to 19.90%.

#### 2.2.4 Applications

We tested nine mAbs using the method established in the study. We found that 10D12 showed significant neutralizing activity (Figure 4F). Excluding the 8G6 mAb with a higher EC50, the remaining eight mAbs samples were tested using pseudotyped virus and authentic neutralization assay. A strong correlation was observed between the EC50 values obtained from both methods. We obtained a linear equation as *y* = 0.4912*x* + 0.0023, *R* ^2^ = 0.9988 (Figure 4G).

Then, to solve the limitations of the traditional PRNT and 96‐well PBNA assay (pseudovirus‐based neutralizing antibody assay) assays with low throughput and inability to achieve automation, we scaled up the neutralizing antibody determination method by increasing the plate size from 96 wells to 384 wells. Then, we used both methods to simultaneously measured 48 human clinical sera, and the results showed that there was no significant difference in ID_50_ value between the two (*p* = 0.29 > 0.05) (Figure 4H).

## 3 DISCUSSION

COVID‐19 is an acute respiratory syndrome that can cause respiratory tract infection in humans. The antibody level after infecting the body can be detected 14 days after infection.^26^ At present, the PRNT test is the gold standard for neutralization of SARS‐Cov‐2, which requires isolated authentic viruses and must be operated under a three‐level biological protection level. The assay period is long, and the risk is high, significantly limits the progress of vaccine, drug, and antibody research.^27^

The pseudovirus system provides a convenient tool for virus research, making it easier to evaluate virus inhibitors, neutralizing antibodies, and immune serums.^4^, ^5^ The pseudovirus can simulate the process of authentic viruses binding to receptors and entering cells. Currently, commonly used enveloped virus pseudovirus systems include HIV lentivirus packaging, VSV, and MLV systems. The HIV lentiviral packaging system used in this study is the most widely used pseudoviral packaging system.^27^ Using the HIV pSG3Δenv backbone plasmid and SARS‐CoV‐2 membrane plasmid to cotransfect 293T eukaryotic expression cells, packaging SARS‐CoV‐2 pseudoviruses. Nie et al. added a chemiluminescence reporter group to the pSG3Δenv plasmid. They successfully constructed HIV systems such as EBOV, MARV, LASV, MERS‐CoV, rabies virus, Chikungunya virus, and Nipah virus. The optimized pseudovirus titer is 100−1000 times higher than without addition.^28^ Wang et al. compared the efficiency of three eukaryotic plasmids, pCMV3.1, pCAG3.1, and pLTR3.1, to express luciferase and found that pCMV3.1 had the highest fluorescence value^29^.

This study reengineered the HIV backbone plasmid and inserted the AmCyan fluorescence reporter gene at the *Hpa*I restriction enzyme cutting site of the SG3Δenv plasmid. Additionally, the CMV strong promoter and Kozak sequence (GCCACC) were introduced before the AmCyan gene, greatly enhancing the expression of the fluorescent protein. The Kozak sequence is a nucleotide sequence located after the cap structure at the 5′ end of eukaryotic mRNA, usually GCCACCAUGG, which can bind to translation initiation factors and mediate the translation initiation of mRNA with a 5′ cap structure.^30^ Then, to further improve the titer of the SARS‐CoV‐2‐S‐AmCyan pseudovirus packaged in this experiment, by removing 21 amino acids from the C‐end of the spike, we observed the positive points were 65‐fold than that of the nontruncated cells. It is reported that the common strategy to enhance the titer of pseudoviruses is achieved through truncating the C‐end of the spike, which can eliminate the interference of heterologous viral glycoproteins during virus formation. This enhances spike protein expression, biological activity, and membrane fusion ability.^31^, ^32^ In addition, the pseudoviruses formed by truncation show consistent results in serum and research compared with the nontruncated viruses.^33^, ^34^ ACE2 is well known as the main receptor of SARS‐CoV‐2.^25^ Spike pseudotyped virus could mimic the cell entry process of SARS‐CoV‐2.^35^ When the ACE2 is overexpressed on the target cell surface, the infectivity of the SARS‐CoV‐2 pseudovirus could be enhanced correspondingly.

Since its discovery in the 19th century, fluorescent proteins have received wide attention and research. Various fluorescent proteins have been found, including blue, cyan, green, yellow, orange, red, and so on. Different types of fluorescent proteins can emit different fluorescence colors under different wavelength excitation. They are widely used in pseudovirus construction based on the stable properties of fluorescent proteins, nontoxicity to cells, convenient vector construction, and direct use in live cell assays.

Several studies have discovered that numerous variations of GFP can showcase increased fluorescence intensity, enhanced photostability, and distinct excitation.^36^, ^37^, ^38^ Uliczka et al.^39^ have shown that variants have higher fluorescence intensity than wild‐type GFP. About 35 times,^39^ the backbone plasmid constructed in this study is the cyan fluorescent protein AmCyan, a new type of improved fluorescent protein under the excitation wavelength of 488 nm. Surrounding with hydrophobic groups prevents fluorescence quenching.^40^

The research established a 96‐well plate neutralization assay using fluorescent pseudotyped viruses with AmCyan reporter in the HIV system. Various parameters were optimized during the development of the neutralization assay, including a cell input of 2.5 × 104 cells/well, a virus input of MOI = 0.025, and incubation and detection times of 1 and 36 h, respectively. Then, methodological validation was conducted, and the method exhibited a strong correlation with the traditional 96‐well chemiluminescent pseudovirus assay, with a formula of *y* = 1.7559*x* + 911.86 and *R* ^2^ = 0.9902. A linear regression relationship with the authentic virus was also validated, with *R* ^2^ = 0.9988. In this study, the cutoff values for human and guinea pigs negative were determined to be 40 and 50, respectively (20.5 and 25.6 IU). Wang et al. established a fluorescence neutralization assay method based on VSV‐GFP pseudovirus, and the cutoff were 10 and 20, respectively (5.18 and 10.36 IU). Although there were differences in the ID50 values of the cutoff values between the two methods, after being calibrated with the NIBSC code of 20/136 and 1000 IU/mL, they displayed a consistent pattern. This enhanced the ability to compare different analytical methods.

The fluorescent pseudovirus neutralization assay has a high safety factor. It saves the operation of adding a substrate during detection. It can directly count the number of positive cells, which significantly saves the cost of test consumables. Additionally, the fluorescence detection instrument Biotek is equipped with a robotic arm to realize automatic detection. Dilution of samples, the addition of pseudoviruses and cells can be achieved with the robotic arm, saving many human resources. Using the 384‐well plate can meet the testing of many samples in a short time. In addition, by modifying the backbone plasmid and membrane plasmid and optimizing the transfection ratio, the packaged high‐titer AmCyan fluorescent pseudovirus improved the stability of neutralizing antibody detection.

However, this study also has some limitations. Although 50 human negative serum samples were employed for specificity determination, only four SARS‐CoV‐2 positive human serum samples were involved in the method validation. Instead, 42 positive guinea pig sera were used to make the comparison between the new and conventional measures. In the future, we would extend the validation of this method by introducing more SARS‐CoV‐2‐positive samples collected from human individuals.

In summary, we have developed a method for testing neutralizing antibodies against SARS‐CoV‐2 using fluorescent pseudovirus in 96‐well plate based on the HIV system. Then, we scaled up the neutralizing antibody determination method by increasing the plate size from 96 wells to 384 wells. The approach offers advantages such as high throughput, straightforward operation, low cost, and automation. This approach can significantly accelerate the progress in advancing vaccines and therapeutics for COVID‐19.

## 4 MATERIALS AND METHODS

### 4.1 Cell and samples

Dulbecco's modified Eagle's medium (DMEM) was the culture medium employed. AF cells (constructed in our laboratory) were generated by stable transfection of the ACE2 receptor and furin protease on 293T cells. During the culturing process, the medium containing 150 µg/mL hygromycin B (Hygromycin B) and 10% FBS (Pansera ES; PAN‐Biotech) was used for selection. ACE2 receptor was stably transfected on 293T and Hela cells, resulting in 293T‐ACE2 cells and Hela‐ACE2 cells, respectively. The culture process involved using 15 µg/mL blasticidin (Blasticidin) for selection. BHK21‐ACE2 cells (purchased from Yunzhou Biotechnology Co., Ltd.) were regularly passaged every 2−4 days using 0.25% trypsin‐EDTA (GIBCO).

*Samples*: Five samples of COVID‐19 convalescent patient serum and 50 samples of human negative sera were obtained from plasma donors at a plasma station in Shandong. *Guinea pig positive sera*: 200−220 g of guinea pigs, which were female, were immunized with 100 µg spike protein adsorbed with an aluminum adjuvant. Forty‐two immunizations were conducted at weeks 0, 2, and 4. Serum samples were collected at week 6 for subsequent experiments. Fifty serum samples from 200−220 g of guinea pigs were obtained as negative control animals. Using RSV DNA plasmid, MERS‐CoV DNA plasmid, and SARS‐CoV DNA plasmid, guinea pigs were immunized, with each guinea pig receiving 200 ng of immunization, immunized twice every 14 days. Fourteen days after the second immunization, serum was collected and stored in a laboratory −80°C freezer.

### 4.2 Plasmid and packaging pseudovirus

For packaging the HIV system pseudovirus carrying SARS‐CoV‐2 spike protein, the pSG3‐△env backbone plasmid was digested with the *Hpa*I enzyme. Using PCR technology, the CMV promoter and Kozak sequence were added to the initiation sequence of the cyan fluorescent protein (AmCyan), resulting in the CMV+Kozak+AmCyan plasmid. It was then ligated with the *Hpa*I‐digested pSG3‐△env backbone plasmid to obtain the pSG3‐△env‐CMV+Kozak+AmCyan backbone plasmid. Using the pcDNA3.1 vector, the D614G variant (accession number: EPI_ISL_766872) was cloned to create the membrane plasmid, which was optimized by modifying its codons. A membrane plasmid of D614G lacking 21 amino acids in the C segment was also constructed. In brief, using Lipofectamine transfection reagent, the packaging pseudovirus was performed by simultaneously transfecting 293T cells with the backbone plasmid and the spike plasmid at a 2:1 ratio. After transfection, the culture medium of 293T cells was changed to DMEM with 2% FBS. After 48 h, the pseudovirus supernatant was harvested and mixed with fresh 2% DMEM. The mixture was then centrifuged at 4°C at 4000 *g* for 20 min, and then centrifuged supernatant was aliquoted and stored at −80°C for later use.

### 4.3 Pseudovirus neutralization

The sample was initially diluted at a ratio of 1:30 and then gradient diluted by a factor of 3. Each well is added with SARS‐CoV‐2 Spike fluorescent pseudovirus with a MOI = 0.025. The incubation is carried out at 37°C 5% CO_2_ for 1 h. In the 96‐well plate, each well is seeded with 2.5 × 104 cells. In the 384‐well plate, each well is seeded with 3.0 × 103 AF cells. After incubating in a 37°C 5% CO_2_ incubator for 36−48 h, the number of AmCyan‐positive cells is counted using Biotek. To assess the level of neutralizing antibodies of samples, the Reed‐Muench method was calculated the median inhibitory dilution (ID50).

### 4.4 Western blotting

The expression of ACE2 in 293T cells, AF cells, ACE2 cells, Hela‐ACE2 cells, and BHK21‐ACE2 was determined using western blotting. After adding the loading buffer, heat at 100°C for 5 min. A 30 µL portion of each sample was subsequently utilized for SDS‐PAGE and western blotting analysis. The primary antibody was a recombinant anti‐ACE2 antibody (Sino Biological; 10108‐R003), rabbit monoclonal at 1/500 dilution; the secondary antibody was goat anti‐rabbit IgG, HRP conjugated at 1/10,000 dilution (Cowin Biotech; CW0103S). In addition, the expression of D614G spike and D614Gdel21 spike protein in 293T cells was also determined. The primary antibody was an anti‐spike poly antibody (immunized guinea pig serum) at 1/500 dilution. The secondary antibody was goat anti‐guinea pig IgG H&L (HRP) (Abcam; ab97155). GAPDH was used as a loading control. The primary antibody was anti‐GAPDH mouse monoclonal antibody (Cowin Biotech; CW0100M), and the secondary antibody was goat anti‐mouse IgG, HRP conjugated (Cowin Biotech; CW0102S).

### 4.5 Statistical analysis

The data were analyzed using GraphPad Prism (8.0) software from GraphPad in San Diego, CA. The values are expressed as means ± standard error of the mean (SEM). An unpaired two‐tailed Student's *t*‐test was employed to compare two sets of data. To statistically analyze multiple sets of data, one or two‐way ANOVA tests and Dunnett's multiple comparisons test were utilized. The presentation of the results includes means § standard deviations (SD). The significance thresholds were set as follows: *p value less than 0.05, **p value less than 0.01, ***p value less than 0.005, and ***p value less than 0.0001.

## AUTHOR CONTRIBUTIONS

Youchun Wang and Weijin Huang revised the manuscript. Ziteng Liang wrote the manuscript and analyzed the experimental data; Ziteng Liang, Jincheng Tong, Shuo Liu, Li Zhang, and Jianhui Nie performed the experiments. Jiajing Wu, Xi Wu, Yuanling Yu, Qiong Lu, and Chenyan Zhao assisted with the animal work. All authors have read and approved the final manuscript.

## CONFLICT OF INTEREST STATEMENT

Jiajing is employed by Beijijng Yunling Biotechnology Co., Ltd., but has no potiential relevant financial or non‐financial interest to disclose. The other authors declare no competing interests.

## ETHICS STATEMENT

The Institute of Translational Medicine, South China University has awarded an ethical certificate to human serum (V1.0, 203301), and written informed consent was obtained from all participants. All animal research approved by the Institutional Animal Care and Use Committee at the National Institutes for Food and Drug Control (NIFDC), with an ethical certificate number of 2020(B) 001.

## Supporting information

Supporting Information

## Data Availability

The data supported the results in this study are available from the corresponding author upon reasonable request.
