# Supplementary material for: Development of a SARS‐CoV‐2 neutralization assay based on a pseudotyped virus using a HIV system
Source: MedComm (2020). 2024 Mar 22;5(4):e517. doi: 10.1002/mco2.517 (PMC10959455; doi:10.1002/mco2.517)
Supplement: Supplementary file 1 — Supporting Information [file MCO2-5-e517-s001.docx]

Development of a SARS-CoV-2 neutralization assay based on a pseudotyped virus using a HIV system

Ziteng Liang^1,2^, Jincheng Tong^2^, Xi Wu^2^, Shuo Liu^3^ , Jiajing Wu^4^, Yuanling Yu^3^, Li Zhang^2^, Chenyan Zhao^2^, Qiong Lu^2^, Jianhui Nie^2^*, Weijin Huang^2^ *and Youchun Wang^1,2,3^*

^1^Chinese Academy of Medical Sciences & Peking Union Medical College, No. 9 Dongdan Santiao, Dongcheng District, 100730 Beijing, China;

^2^Division of HIV/AIDS and Sex-transmitted Virus Vaccines, Institute for Biological Product Control, National Institutes for Food and Drug Control (NIFDC), WHO Collaborating Center for Standardization and Evaluation of Biologicals, NHC Key Laboratory of Research on Quality and Standardization of Biotech Products and NMPA Key Laboratory for Quality Research and Evaluation of Biological Products, 102629 Beijing, China;

^3^Changping Laboratory, Yard 28, Science Park Road, Changping District, Beijing, China;

^4^Beijing Yunling Biotechnology Co., Ltd., Beijing 100176, China.

*****Correspondence: Jianhui Nie: [niejianhui@nifdc.org.cn](mailto:niejianhui@nifdc.org.cn), Weijin Huang: huangweijin@nifdc.org.cn or Youchun Wang[: wangyc@nifdc.org.cn](mailto:(wangyc@nifdc.org.cn))

**Supplementary Material**


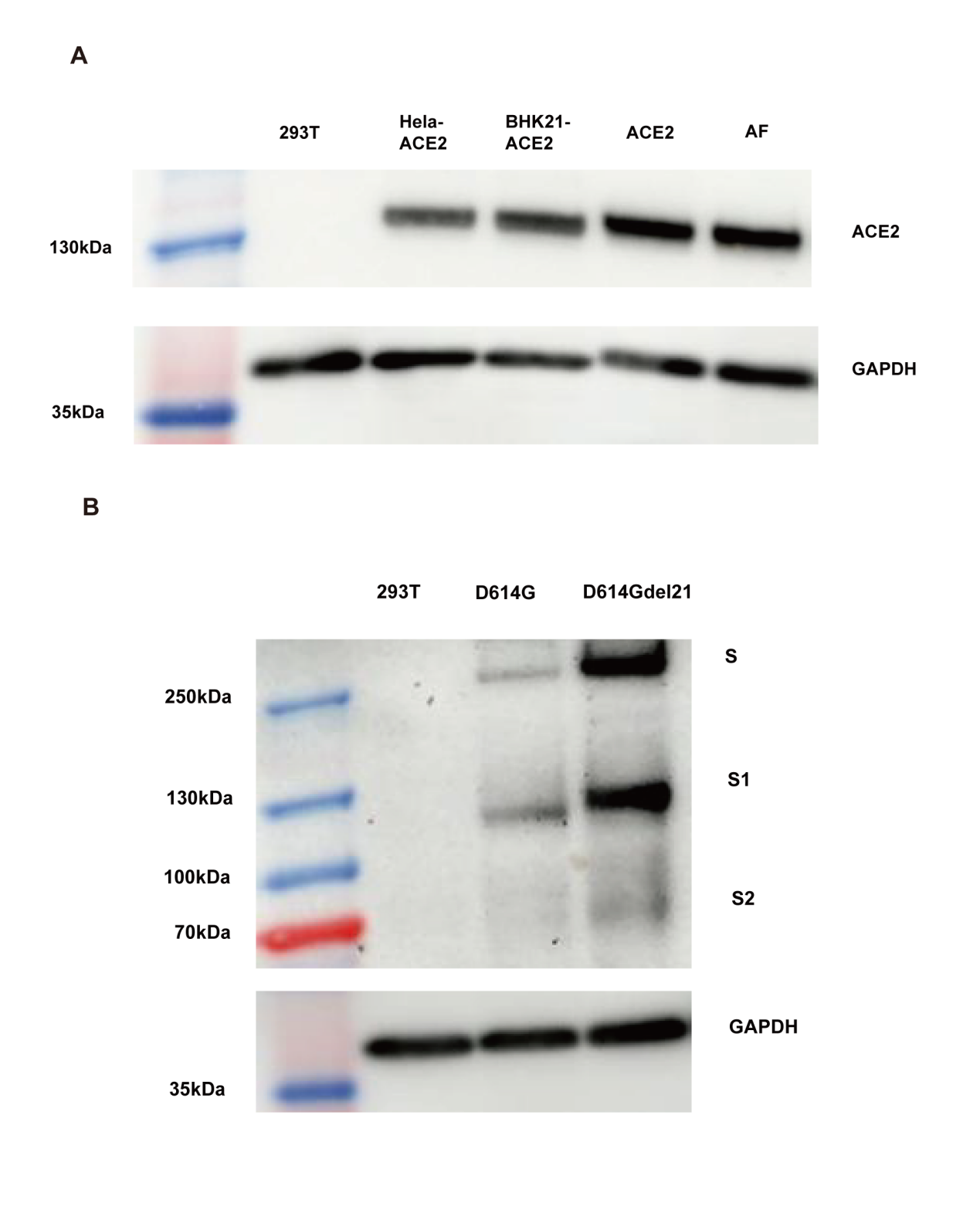


Figure S1. Expression of ACE2 and spike protein using Western blotting. (A) Expression of ACE2 in 293T cells, AF cells, ACE2 cells, Hela-ACE2 cells and BHK21-ACE2 were determined using Western blotting. (B) Expression of D614G spike and D614Gdel21 protein in 293T cells using Western blotting.
